# Appalachian disparities in tobacco cessation treatment utilization in Medicaid

**DOI:** 10.1186/s13011-020-0251-0

**Published:** 2020-01-20

**Authors:** Amie Goodin, Jeffery Talbert, Patricia R. Freeman, Ellen J. Hahn, Amanda Fallin-Bennett

**Affiliations:** 1 University of Florida, College of Pharmacy, Gainesville, FL USA; 2 Center for Drug Evaluation and Safety (CoDES), Gainesville, FL USA; 3 University of Kentucky, College of Pharmacy, Lexington, KY USA; 4 University of Kentucky, College of Nursing, Lexington, KY USA

**Keywords:** Tobacco cessation, Medicaid, Affordable care act

## Abstract

**Background:**

Kentucky Medicaid enrollees, particularly those in the rural Appalachian region, face disproportionate smoking rates and tobacco-related disease burden relative to the rest of the United States (US). The Affordable Care Act (ACA) mandated tobacco cessation treatment coverage by the US public health insurance program Medicaid. Medicaid coverage was also expanded in Kentucky, in 2013, with laxer income eligibility requirements. This short report describes tobacco use incidence and tobacco cessation treatment utilization, comparing by Appalachian status before and after ACA-mandated cessation treatment coverage.

**Methods:**

The study design was a retrospective cross-sectional analysis from 2013 to 2015. Subjects were Medicaid enrollees with 1) diagnosis of any tobacco use (2013 *n* = 541,349; 2014 *n* = 864,183; 2015 *n* = 1,090,274); and/or (2) procedure claim for tobacco cessation counseling, and/or (3) pharmaceutical claim for varenicline or any nicotine replacement product. Primary measures included tobacco use incidence and proportion of users receiving cessation treatment. Analysis was via chi square testing of change by year.

**Results:**

Overall, the proportion of tobacco users utilizing cessation treatment decreased (4.75% tobacco users in 2013; 3.15% in 2015). Tobacco users receiving counseling decreased from 2.06% pre-ACA (2013) to 1.06% post-ACA (2015, *p* < 0.001), as did the proportion receiving nicotine replacement products post-ACA (2.69% in 2013 to 1.55% by 2015; *p* < 0.001). More Appalachians received cessation treatment than non-Appalachians in 2013 (2.72% vs. 2.03%), but by 2015 non-Appalachians received more treatment overall (1.50% vs. 1.65%; *p* < 0.001). Appalachians received more counseling and NRT, but less varenicline, than non-Appalachians.

**Conclusions:**

Utilization of all forms of tobacco cessation treatment throughout Kentucky, and particularly in rural Appalachia, remained limited despite Medicaid enrollment as well as coverage expansions. These findings suggest that barriers persist in access to tobacco cessation treatment for individuals in Medicaid.

## Background

Tobacco use is the leading cause of preventable death and disease, contributing to 480,000 deaths annually [1]. While smoking rates have declined across the United States, certain states, including Kentucky, experience disproportionate burden. Kentucky leads the United States in lung cancer cases, with a rate of 92.4 per 100,000 persons compared to the national rate of 60.4 per 100,000 [2]. In addition, Kentucky Medicaid enrollees [3] and those in the rural Appalachian region have particularly high smoking rates [4], and face a high burden of tobacco-related morbidity and mortality. Appalachian Kentucky has among the highest cardiopulmonary disease and cancer rates in the U.S. [4, 5].

Increased availability of tobacco treatment services could reduce tobacco use, leading to improved chronic disease outcomes. The 2010 Patient Protection and Affordable Care Act (ACA) expanded Medicaid eligibility to include individuals at 138% of the federal poverty level. Starting in 2014, Medicaid health plans were required to cover two quit attempts per year, with each quit attempt consisting of four counseling sessions (comprised of individual sessions with the provider) and 90-day supplies of any Food and Drug Administration approved cessation medication or nicotine replacement therapy product. Prior to this 2014 change, Medicaid plans in this state were not required to cover counseling (except for pregnant women), cessation medications, nor nicotine replacement therapy products [6]. Additionally, Medicaid did not permit plans (fee for service or managed care) to impose lifetime quit attempt limits and did not permit stepped-care therapy restrictions in this state starting in 2014 [7]. These expansions in cessation treatment coverage requirements were enacted simultaneously with Medicaid expansion in Kentucky. It should be noted that some Medicaid plans administered by Managed Care Organizations (MCOs) did cover some tobacco cessation treatment options prior to 2014, but this coverage was both inconsistent and non-comprehensive.

Given the potential change in access to tobacco cessation treatment brought about by Medicaid enrollment expansion and increased coverage of medical services and prescriptions for tobacco cessation treatment, the study aims were to: (1) describe incidence of tobacco use and tobacco cessation treatment utilization over time, from pre- to post-Medicaid expansion in Appalachian and non-Appalachian regions of Kentucky; (2) compare utilization of tobacco cessation treatment pre−/post-expansion by demographic characteristics and by Appalachian status.

## Methods

### Design

This study is a cross-sectional, retrospective analysis.

### Setting and sample

The sample included all Kentucky Medicaid enrollees who met one or more of the following conditions within any year from 2013 to 2015: 1) a diagnosis of any Tobacco Use or related Disorder (International Disease Classifications [ICD] versions 9 and 10; 305.1, 649.0x, 989.84, V1582); 2) a procedure claim for tobacco cessation treatment counseling (procedure codes 99,406–99,407); and/or 3) a pharmaceutical claim for a tobacco cessation medication (varenicline) or a nicotine replacement (NRT) product (i.e., nicotine gum, lozenge, patch, or nicotine inhaler; a complete list of National Drug Codes is available as Additional file 1: Table S1). An individual could be represented among each of the years in the study period.

### Measures

Indicators for Medicaid enrollee characteristics included gender, and county of residence, which was used to classify enrollees as Appalachian residents, according to Appalachian Regional Commission definitions. Primary measures were tobacco use incidence and tobacco cessation treatment utilization, which was defined as presence of medical and/or prescription claim for any of the following: cessation counseling, varenicline prescription, NRT prescription.

### Statistical analysis

Tobacco user incidence was calculated for each year in the study period as a proportion of all Medicaid enrollees based on the presence of a tobacco use diagnosis. Tobacco cessation treatment utilization was calculated as number and percent of diagnosed tobacco users for all Medicaid enrollees and for only tobacco users. Change over time in total tobacco cessation treatment utilization was analyzed descriptively by year as a proportion of tobacco users, and then stratified by enrollee characteristics. Tobacco cessation treatment utilization was reported for both number of unique individual tobacco users with claim or service for any cessation product or service, and number of unique individual tobacco users by type of cessation service or claim in each calendar year. Medicaid enrollees were assigned unique identifiers within each calendar year, so trends in individual enrollee uptake of tobacco cessation treatment was not assessed over time. Chi square analysis was used to test for differences in characteristics of tobacco users and cessation treatment utilization for 2013 (pre-expansion) versus 2014 and 2015 (post-expansion), with a priori significance set at alpha = 0.05. Analyses were conducted in Stata v12.0. This study was deemed exempt from Institutional Review Board review due to the absence of identifying information.

## Results

In 2013, 52.11% of Kentucky Medicaid enrollees had at least one tobacco use diagnosis and by 2015 this increased to 63.97% (Table 1). Overall utilization of tobacco cessation treatment was low throughout the study period, with 4.75% of tobacco users receiving any cessation treatment in 2013 and 3.15% receiving any cessation treatment in 2015. The most common type of cessation treatment received in all years was counseling, which has been pulled out separately in Table 1 from all other products and services (varenicline and NRT). However, the proportion of tobacco users receiving counseling decreased from 2.06% in 2013 (pre-expansion) to 1.06% by 2015 in the post-expansion period (*p* < 0.001), as did the proportion receiving nicotine replacement products and/or varenicline post-expansion (2.96% in 2013 to 1.95% by 2015; p < 0.001). Fewer than 1% of tobacco users received the cessation medication varenicline during any year in the study period, and the proportion of cessation medication recipients decreased significantly in the post-expansion period (0.71% in 2013 to 0.41% by 2015; *p* < 0.001).

**Table 1:** Characteristics of Tobacco Users Receiving Cessation Treatment in Kentucky Medicaid, pre- and post-Medicaid Expansion

	2013 (Pre-Expansion)	2014 (Post-Expansion)	2015 (Post-Expansion)	Pre- vs. Post- Expansion
Tobacco User Characteristic	*n (%)*	*n (%)*	*n (%)*	*p-value*
Gender				
Female	376,840 (69.61%)	511,616 (59.20%)	641,257 (58.82%)	< 0.001*
Male	164,509 (30.39%)	352,567 (40.80%)	449,017 (41.18%)	
Appalachian Residence				
Appalachian County	257,817 (47.62%)	340,210 (39.37%)	397,022 (36.41%)	< 0.001*
Non-Appalachian County	283,532 (52.38%)	523,973 (60.63%)	693,252 (63.59%)	
Total Tobacco Users	541,349	864,183	1,090,274	
Total Enrollees (% Tobacco Users)	1,038,766 (52.11%)	1,540,648 (56.09%)	1,704,472 (63.97%)	
Received Any Tobacco Cessation	*n (% Tobacco Users)*	*n (% Tobacco Users)*	*n (% Tobacco Users)*	*p-value*
Gender				
Female	17,238 (3.18%)	16,331 (1.89%)	20,995 (1.93%)	< 0.001*
Male	8481 (1.57%)	10,206 (1.18%)	13,331 (1.22%)	
Appalachian Residence				
Appalachian County	14,725 (2.72%)	13,728 (1.59%)	16,359 (1.50%)	< 0.001*
Non-Appalachian County	10,994 (2.03%)	12,809 (1.48%)	17,967 (1.65%)	
Type of Tobacco Cessation				
Counseling Services	11,138 (2.06%)	13,829 (1.60%)	17,403 (1.60%)	< 0.001*
Nicotine Replacement Products or Varenicline	14,581 (2.69%)	12,708 (1.47%)	16,923 (1.55%)	< 0.001*
Total Receiving Any Tobacco Cessation Treatment (% Tobacco Users)	25,719 (4.75%)	26,537 (3.07%)	34,326 (3.15%)	
% Enrollees Receiving Any Tobacco Cessation Treatment	2.48%	1.72%	2.01%	

*Indicates statistical significance

There were also significant changes in the tobacco-using enrollee population demographics during this period, with an influx of male tobacco users post-expansion (from 30.39% of users in 2013 to 41.18% by 2015; *p* < 0.001). Enrollment in Medicaid in general experienced a significant increase during the expansion, with an increase from 1.03 million Medicaid enrollees in 2013 to over 1.7 million Medicaid enrollees by 2015. Appalachians comprised a smaller proportion of total Kentucky Medicaid tobacco users by 2015 than in 2013.

More Appalachian residents received tobacco cessation treatment overall than non-Appalachians in 2013 (2.72% Appalachian; 2.03% non-Appalachian), but by 2015 non-Appalachians received more tobacco cessation treatment (1.50% Appalachian; 1.65% non-Appalachian; *p* < 0.001). When examining type of tobacco cessation treatment used by residency status, tobacco cessation medication use (varenicline) in Appalachia was rare, with only 0.63% of Appalachian tobacco users receiving the cessation medication varenicline in 2013, 0.38% in 2014, and 0.40% by 2015 (Table 2).

**Table 2:** Tobacco Cessation Treatment Utilization by Appalachian Residence Status in Kentucky Medicaid, pre- (2013) and post-Medicaid Expansion (2014–2015)

	2013 (Pre-Expansion)	2014 (Post-Expansion)	2015 (Post-Expansion)	Pre- vs. Post- Expansion
Cessation Medication (varenicline)	*n (% Tobacco Users)*	*n (% Tobacco Users)*	*n (% Tobacco Users)*	*p-value*
Appalachian Resident	1615 (0.63%)	1298 (0.38%)	1572 (0.40%)	< 0.001*
Non-Appalachian Resident	2208 (0.78%)	2428 (0.46%)	2868 (0.41%)	
Cessation Counseling Services				
Appalachian Resident	7677 (2.98%)	8363 (2.46%)	9053 (2.28%)	< 0.001*
Non-Appalachian Resident	3461 (1.22%)	5466 (1.04%)	8350 (1.20%)	
Nicotine Replacement Products				
Appalachian Resident	5433 (2.11%)	4067 (1.20%)	5734 (1.44%)	< 0.001*
Non-Appalachian Resident	5325 (1.88%)	4915 (0.94%)	6749 (0.97%)	

*Indicates statistical significance

## Discussion

The ACA and Medicaid expansion presented an opportunity to improve access to evidence based tobacco treatment [8]; access is associated with tobacco quit attempts and cessation [9]. However, results of this study indicate that overall tobacco cessation treatment utilization remained low among Kentucky Medicaid in Appalachian and non-Appalachian enrollees through 2015. This was likely due to implementation barriers such as lack of information about covered services, inconsistent formularies across plans, and requirements for prior authorization [10]. Burdensome prior authorization requirements are particularly implicated in this state as previous reports have indicated that other common barriers to tobacco cessation treatment, such as lifetime limits on quit attempt coverage and sporadic implementation of cost-sharing removal, were not present in this state in 2014 and 2015 after the ACA change [7]. However, limited patient readiness to change and low demand for tobacco cessation treatment have also been implicated in prior studies [11], which could contribute to the low utilization after Medicaid expansion that was observed in these findings.

Study findings also support a body of literature that healthcare providers often fail to treat tobacco dependence [12–14]. This is due to a variety of barriers including time constraints, lack of training in tobacco dependence and treatment, and provider pessimism about their ability to help their patients stop using tobacco as well as perceived patient resistance [14]. The low utilization of evidence based tobacco treatment despite increased coverage supports the need for widespread change in the delivery of tobacco treatment. One approach would be shifting from the current opt-in tobacco treatment approach (using readiness to quit as a precondition) to an opt-out strategy (offering evidence based tobacco treatment to all smokers) [15]. In the Appalachian region, however, strategies would likely need further tailoring to first address regional barriers, such as persistent shortages in primary care [16, 17].

Although there was only a slight increase in uptake in tobacco cessation treatment, the characteristics of Kentucky Medicaid tobacco users changed following Medicaid expansion, with a greater proportion of males receiving tobacco use diagnoses than before the expansion. Concerningly, Appalachian tobacco users comprised 36% of all Kentucky Medicaid tobacco users by 2015 in the post-ACA period. Given that the total population of the Appalachian region comprises fewer than 25% of the state’s total population [18], this suggests that Appalachia continues to face disparate tobacco use burden.

Overall Medicaid enrollment pre-ACA expansion (2013) to the end of the study period (2015) more than doubled and it is possible that individuals who qualified under the expansion eligibility requirements were inherently different than individuals who qualified under the more stringent pre-ACA expansion eligibility criteria. Owing to this limitation, our analysis intended to compare cross-sectional snapshots across pre- and post-expansion calendar years and is not a longitudinal study design. It should also be considered that Medicaid eligibility (and therefore enrollment) was more volatile in the pre-ACA expansion period, with one study estimating that more than half of Medicaid enrollees in this state were subject to at least one period of both eligibility and ineligibility within a single calendar year [19]. It is unclear whether the Appalachian region was impacted by ACA-expansion differently than other regions in the state; though, our analysis suggests that enrollment increases in Appalachia were not as significant as enrollment increases elsewhere.

It should also be noted that our 2013 tobacco use estimates vary from Ku et al.’s analysis in which prevalence was estimated to be 40% in 2013 [20]; however, their analysis relied on extrapolated survey data and not on diagnoses. Although Appalachian enrollees had higher utilization of tobacco cessation counseling and NRT, use of varenicline medication in Appalachia remained particularly rare. Promotion of tobacco cessation medications such as varenicline in Appalachia has promise as a strategy to reduce the staggering tobacco-related disparities in the region.

There are several limitations in the analysis. First, only diagnoses within the year of the qualifying diagnosis, procedure, or pharmaceutical claim were available for each enrollee, so prior diagnoses of tobacco use disorder were not accounted for. Second, tobacco use incidence in Medicaid enrollees in this state in 2013 was lower in another analysis (40% in 2013) than the incidence calculated in this study [20], which suggests that reliance on diagnosis codes to identify tobacco users may be subject to misclassification of current smoking status. In particular, we used diagnoses codes for “history of tobacco use” in addition to codes that specify current tobacco use and/or nicotine dependence to improve sensitivity of capture, but this strategy may result in overestimation of current smoking status due to inability to disentangle true incident cases from year to year. Third, data on any cessation treatment that the provider did not bill for or that the enrollee paid for in cash was not available. The analysis was not stratified by traditional Medicaid enrollment (fee for service) versus Managed Care Organization (MCO) plan enrollment due to > 90% MCO penetration in this state [21]. Finally, we did not examine claims for bupropion (Wellbutrin®, Zyban®), because the medication is both a smoking cessation aid and an antidepressant and we were not able to directly assign the indication for the pharmaceutical claim via secondary analysis of claims. This exclusion would lead to underestimation of overall tobacco cessation treatment medication utilization, which is why cessation medications are explicitly referred to as formulations of varenicline.

## Conclusions

In the wake of expanded Medicaid enrollment and a consistently high proportion of tobacco users, tobacco cessation treatment utilization among Kentucky Medicaid enrollees remained low despite the implementation of mandated tobacco cessation coverage. Future studies are needed to assess and resolve barriers to the provision of tobacco cessation treatment, particularly for the Appalachian region that faces persistent health disparities and poorer access to care.

## Supplementary information

**Additional file 1: Table S1.** National Drug Codes (NDC) for Varenicline. This supplementary table includes the full list of national drug codes for all varenicline formulations. These codes were used to identify the tobacco cessation medication of interest, varenicline, in pharmacy claims from Medicaid.

## Data Availability

The datasets analyzed during this study are not publicly available due to terms specified by the data use agreement with Kentucky Cabinet for Health and Family Services, Department of Medicaid Services.

## Abbreviations

ACA: Patient Protection and Affordable Care Act
MCO: Managed Care Organization
NRT: Nicotine Replacement Therapy
