# Supplementary material for: Appalachian disparities in tobacco cessation treatment utilization in Medicaid
Source: Subst Abuse Treat Prev Policy. 2020 Jan 20;15:5. doi: 10.1186/s13011-020-0251-0 (PMC6971922; doi:10.1186/s13011-020-0251-0)
Supplement: Supplementary file 1 — Additional file 1: Table S1. National Drug Codes (NDC) for Varenicline. This supplementary table includes the full list of national drug codes for all varenicline formulations. These codes were used to identify the tobacco cessation medication of interest, varenicline, in pharmacy claims from Medicaid. [file 13011_2020_251_MOESM1_ESM.docx]

**Additional file 1: Table S1. National Drug Codes (NDC) for Varenicline**

| 63539-473-10 |
| --- |
| 0069-0468-56 |
| 0069-0469-03 |
| 0069-0469-11 |
| 0069-0469-12 |
| 0069-0469-56 |
| 0069-0471-01 |
| 0069-0471-02 |
| 0069-0471-03 |
| 35356-012-56 |
| 35356-174-56 |
| 35356-175-53 |
| 54868-5664-0 |
| 54868-5674-0 |
| 21695-945-53 |
| 21695-946-56 |
| 63187-618-53 |
| 55154-2728-0 |
| 55154-2728-4 |
| 55154-2728-6 |
| 68151-4953-5 |
| 68151-4952-5 |
| 35356-011-07 |
| 35356-011-14 |
| 35356-011-56 |
| 21695-633-56 |
